# Photonics tools begin to clarify astrocyte calcium transients

**DOI:** 10.1117/1.NPh.9.2.021907

**Published:** 2022-02-18

**Authors:** Kelsea A. Gorzo, Grant R. Gordon

**Affiliations:** University of Calgary, Hotchkiss Brain Institute, Cumming School of Medicine, Calgary, Alberta, Canada

**Keywords:** astrocyte, calcium, stimulation emission depletion, genetically encoded fluorescent calcium indicator, two-photon, analysis

## Abstract

Astrocytes integrate information from neurons and the microvasculature to coordinate brain activity and metabolism. Using a variety of calcium-dependent cellular mechanisms, these cells impact numerous aspects of neurophysiology in health and disease. Astrocyte calcium signaling is highly diverse, with complex spatiotemporal features. Here, we review astrocyte calcium dynamics and the optical imaging tools used to measure and analyze these events. We briefly cover historical calcium measurements, followed by our current understanding of how calcium transients relate to the structure of astrocytes. We then explore newer photonics tools including super-resolution techniques and genetically encoded calcium indicators targeted to specific cellular compartments and how these have been applied to astrocyte biology. Finally, we provide a brief overview of analysis software used to accurately quantify the data and ultimately aid in our interpretation of the various functions of astrocyte calcium transients.

## 1 Introduction

The past 30 years have witnessed a dramatic reappraisal of the role astrocytes play in the brain. With a central position in the neurovascular unit, each astrocyte is tasked with integrating information from both neurons and the microvasculature to coordinate brain activity and metabolism. As such, new compelling evidence demonstrates previously unimagined roles for these cells in many aspects of neuro(patho)physiology. Many investigators are recognizing the importance of studying astrocytes and the diverse effects these cells exert on synapses, mural cells, endothelial cells, and other glial targets.^1^^–^^3^ It is believed that a primary mechanism through which astrocytes transduce and encode information is by elevations in intracellular calcium.^4^ These calcium events are incredibly diverse, varying in area, amplitude, duration, propagation direction, and compartmental localization. Furthermore, calcium transients can be evoked by various physiological changes, synaptic activity, pathological events, or simply occur spontaneously.^5^ These calcium signals occur throughout different compartments of the cell. Astrocytes have complex morphology in which the majority of the cell volume (up to 85%)^6^^,^^7^ is comprised of irregular, nanoscopic (30 to 50 nm) processes that sit below the diffraction limit of light.^8^ As such, the implementation of new microscopy and labeling approaches has been necessary to tease apart different calcium sources. Understanding how changes in astrocyte calcium drive communication at the neuronal–glial–vascular interface is a vibrant area of research; however, its success is closely linked to the availability and efficacy of fluorescent indicators, optical imaging methods, and analysis tools.

In this review, we cover measurements of calcium dynamics in astrocytes using optical imaging tools. First, we briefly describe how calcium signaling emerged as an important player in neuronal–astrocyte communication and glial–vascular communication, followed by an outline of our current understanding of calcium transients in relationship to the structure of astrocytes. Then, we delve into the tools used to accomplish this, spanning from the development of calcium-specific, small molecule dyes up to the current use of genetically encoded calcium indicators (GECIs). We then review microscopy methods particularly well suited to astrocyte calcium imaging and conclude with describing the latest analysis software, which will be vital to decoding the biological relevance of calcium transients in astrocytes.

## 2 Importance of Astrocyte Calcium Emerges

An important clue pointing to previously undiscovered roles of astrocytes in neurophysiology came with the finding that astrocytes express glutamate-sensitive ion channels.^9^ Following this, the seminal discovery of Cornell-Bell et al.^10^ demonstrated that bath application of glutamate induces calcium waves in the cytoplasm of astrocytes introducing the potential link between neural activity and calcium transients. This phenomenon was later confirmed using stimulation protocols in slice^11^^,^^12^ and then later *in vivo* via sensory stimulation in awake animals.^13^ Following this, the next step in recognizing fluctuations in astrocyte calcium as an extra neuronal signaling system came with the discovery that these changes regulate the release of key molecules such as ATP,^14^ lactate,^15^ or hormones such as atrial natriuretic peptide.^16^ These effectors exert powerful changes not only on neurons to modulate synaptic transmission^17^ and plasticity^18^ but also influence other targets including the mural cells (smooth muscle cells and pericytes) of vasculature to control the diameter of cerebral blood vessels and thereby blood flow.^19^^–^^22^

## 3 Complexities of Astrocyte Morphology

The unique star-like morphology of astrocytes allows the many processes of these cells to come in close proximity to important targets, such as the synapse or microvasculature.^23^ Depending on the physical location in the astrocyte, distinct arrays of ion channels, organelles, and cellular machinery are present allowing for specialized and compartmentalized functions.^5^ Commonly referred to as a microdomain, these spatially restricted areas where calcium transients occur were first discovered in Bergmann glia.^24^ Typically, calcium microdomains describe events restricted to the ultrafine processes of the astrocyte; however, common nomenclature precisely describing the structures in which a microdomain exist is somewhat absent. Most recently, Verkhratsky and Semyanov suggest a classification system based on morphological properties of the astrocyte arbor where branches and branchlets describe the main astrocytes processes originating from the soma (resolvable with diffraction limited optical microscopy), which then split into higher order branchlets or fine leaflets, the latter of which are below the diffraction limit, thereby unresolvable with traditional microscopes.^25^ Other terms such as perisynaptic astrocyte processes (PAPs)^6^ along with nodes and shafts^26^ have been used to describe the nanoscopic processes of astrocytes. These are all separate from a major process arising from the soma, at least one of which becomes a specialized endfoot covering a nearby blood vessel. Endfeet come in a variety of shapes, thicknesses, and sizes, partially reflecting whether the endfoot is covering an arteriole, venule, or capillary.^27^

Distinguishing the structural complexity of astrocytes particularly in the context of localizing calcium transients in fine processes requires innovative imaging approaches.^7^ Three-dimensional (3D) reconstructions of tissue sections using electron microscopy (EM) has provided intricate detail of PAPs^28^^–^^31^ and endfeet.^27^^,^^32^^,^^33^ However, a significant limitation of this approach is that fixation of the tissue is required prior to imaging, thereby limiting experimental flexibility. To capture the dynamic nature of PAPs and endfeet *in situ* or *in vivo*, recent studies have looked to super-resolution microscopy methods, which are compatible with live tissue samples, such as two photon stimulation emission depletion (2P-STED). Tools such as 2P-STED provide us with the ability to examine the interactions between nanostructures under physiologically relevant states.

## 4 Sources of Astrocyte Calcium

Teasing apart the physiological role of astrocyte calcium signaling is highly dependent on the physical location in which the calcium events are occurring, the membrane protein mediating the calcium flux and the downstream pathways. The soma, branches, and leaflets all possess unique cellular machinery that can generate and respond to calcium elevations, which are often completely dissociated from fluctuations occurring simultaneously in other parts of the same cell.^5^ Typically, calcium transients in the astrocyte arbor are more frequent and appear to be produced by a wider variety of cellular components. Given a very high surface area-to-volume ratio, it was previously believed that no organelles were present in the cytosol of PAPs. However, serial EM revealed that 40% of cortical PAPs have at least one organelle (endoplasmic reticulum [ER] or mitochondria).^34^ Deletion of the ER IP_3_ receptor type 2 (IP_3_R2), the common effector for IP_3_ signaling initiated by G-protein coupled receptors activity in astrocytes,^35^ results in a significant reduction (∼60%) in the frequency and amplitude of spontaneous calcium events in the astrocyte arbor.^36^^–^^40^ It is important to note that IP_3_Rs are also found in specialized astrocyte endfeet that encase cerebral arterioles and may play an important role in coordinating cerebral blood flow with the metabolic demand of neurons. IP_3_ released from perisynaptic processes can initiate an IP_3_R-dependent calcium wave that propagates through the astrocyte to the endfoot, where the increase in calcium initiates the release of vasoactive molecules, causing vasodilation.^41^ In terms of detecting neuronal activity, metabotropic glutamate receptors (mGluRs) on astrocytes, commonly mGluR5, coupled to $G_{q/11}$ proteins stimulate IP_3_ production.^42^^,^^43^ However, variations in the presence of mGluRs in the young versus old brain have been demonstrated. Sun et al.^44^ report mGluR5 expression significantly decreases with age, to such a point where pharmacological activation fails to produce somatic IP_3_-mediated calcium increases. Other studies show calcium elevations in mature astrocytes via whisker stimulation are mediated through synaptic glutamate release, resulting in astrocyte mGluR5 activity^13^ that is localized to PAPs.^17^^,^^45^ A plausible explanation for this discrepancy could be attributed to the focus of Sun et al. upon somatic calcium responses, which likely differ from those in the processes of astrocytes. Furthermore, as the brain matures and refinement of synaptic circuitry takes place, it is possible that confinement in the distribution of mGluRs occurs to PAPs, where they are required for localized modulation at the tripartite synapse.^1^^,^^46^ Other than glutamate, numerous G protein coupled receptors such as $G_{i}$ protein ${\mathrm{GABA}}_{B}$ receptors, $G_{i}$ protein CB1 receptors, or $G_{q}$ protein alpha adrenergic receptors can also cause increases in calcium via convergence on an ${\mathrm{IP}}_{3}R$ signaling pathway.^47^^–^^51^

Although calcium transients rely in part on release from intracellular stores, influx of calcium through transmembrane channels or transporters is not to be overlooked.^52^ Neurotransmitter transporters such as GLT-1 or GAT-3 can generate increases in intracellular sodium during cotransport, which ultimately reverses the activity of the ${\mathrm{Na}}^{+}/{\mathrm{Ca}}^{2+}$ exchanger (NCX), resulting in calcium influx.^53^^–^^55^ This effect was demonstrated with bath application of GABA.^56^ It also has been observed that TRPA1 generates frequent calcium influxes, localized close to the plasma membrane, but this observation has been contested.^52^ Shigetomi et al.^57^ do however, provide evidence that TRPA1 in maintains basal calcium levels and regulates d-serine release into the extracellular space. Similarly, TRPC1, another commonly expressed cation channel in astrocytes, has been shown to be responsible for replenishing internal calcium stores, in addition to driving calcium-dependent glutamate release.^58^

Other interesting possible sources of transmembrane calcium transients occur upon activation of mechanosensitive channels. Positioned around cerebral arterioles, astrocyte endfeet acts as intracranial baroreceptors that sense changes in perfusion via fluctuations in the vascular lumen diameter.^59^^–^^61^ This mechanosensory signaling is thought to be mediated through pannexin 1 (Panx1) hemichannels,^62^ PIEZOs,^63^ and TRPV4.^61^^,^^64^ Turovsky et al.^64^ demonstrated that TRPV4 in particular contributes to mechanical gating of Cx43 hemichannels, resulting in the release of ATP, which amplifies calcium signaling via P2Y1 receptors and recruitment of intracellular calcium stores. Haidey et al. observed that arteriole vasoconstriction elevates endfoot calcium via TRPV4, which immediately engages a feedback dilation pathway to constrain the extent of constriction. *In vivo*, this pathway limited the amplitude of rhythmic fluctuations in arteriole diameter.^61^

Recent reports have also connected mechanical changes of PAPs to calcium signaling in response to LTP induction. PAPs, which are positioned adjacent to synapses and rich in GLT-1 transporters, are responsible for containing glutamate to the synaptic cleft. If substantial “spillover” occurs, there can be significant physiological impacts including NMDA receptor-mediated intersynaptic crosstalk, heterosynaptic potentiation, and remote activation of mGluRs. Rusakov’s team demonstrate using super resolution imaging that PAPs withdraw from potentiated synapses following LTP.^65^ Interestingly, while this group believes this process to be mediated by NKCC1, others have found astrocytic calcium signaling more central to this process. Jones et al. overexpressed mGluR1/5, which upon stimulation increased PAP coverage of synapses and directly affected the long-term survival of these spines.^66^ Given that PAPs contain numerous actin-related proteins,^45^ Molotkov et al.^67^ found that calcium-dependent actin binding protein, profilin-1 is essential to PAP plasticity. Further research is required to functionally link synaptic activity with modulation of the cytoskeleton. Nevertheless, the structural changes of PAPs surely influence calcium dynamics, likely impacting many important downstream processes.

## 5 Calcium Indicators

**Fig. 1:**
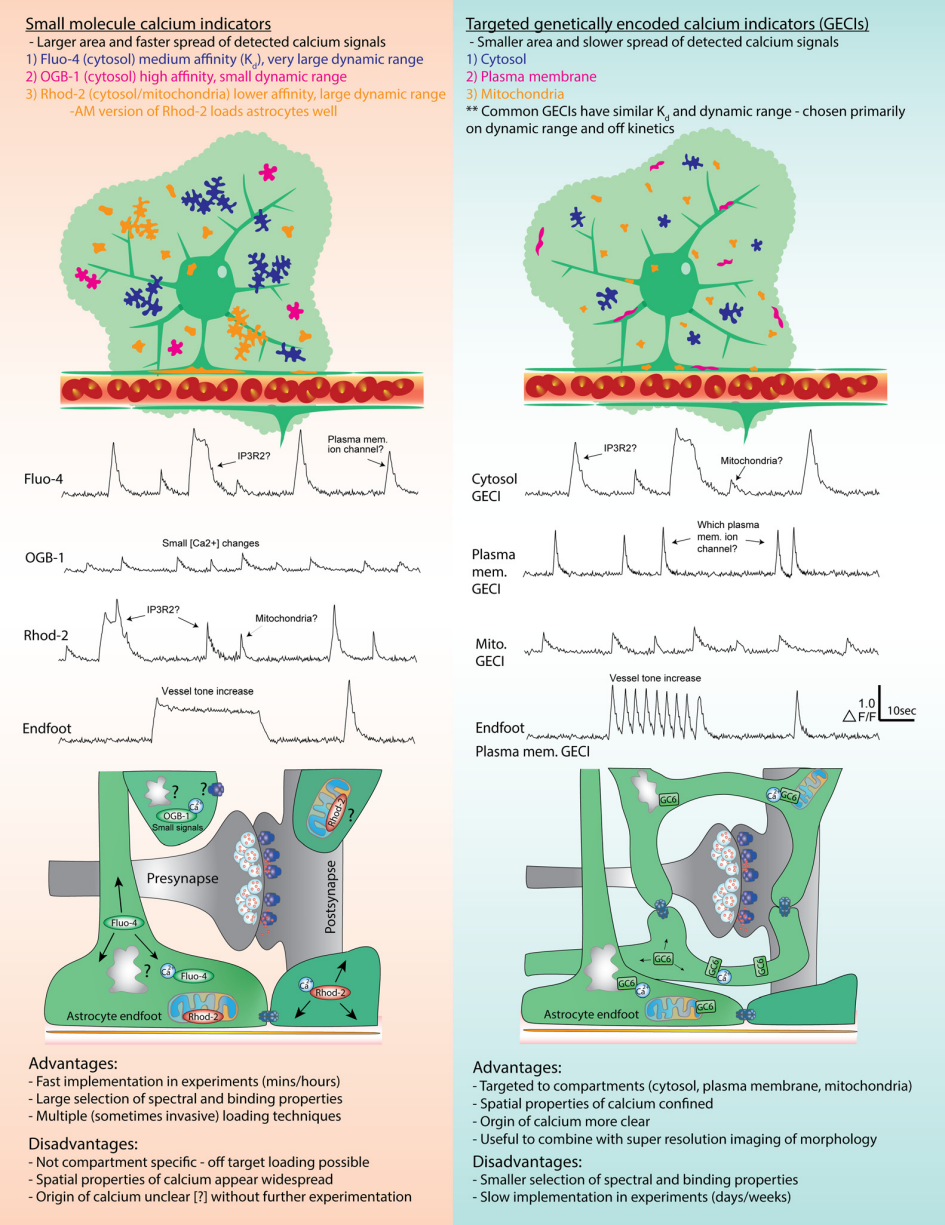
A comparison of small molecule calcium indicators with GECIs. A visual representation of the area of detected calcium signals inside the astrocyte is shown along with representative ΔF/F traces and general properties for various common indicators. A description of the advantages and disadvantages for small molecule indicators versus GECIs is also provided.

Significant advancements in the biotechnology underpinning fluorescent calcium indicators continue to drive our understanding of the complex role calcium plays in neurophysiology. The ability to measure intracellular calcium originates with small molecule dyes that consisted of a calcium chelating complex conjugated to a fluorescent reporter.^68^^,^^69^ Fura-2, which was one of the first widely utilized indicators, demonstrates a shift in excitation spectra in the presence of calcium therefore the ratio of fluorescence emitted at two different excitation wavelengths can be used to determine the concentration of calcium.^70^ More recently, studies investigating rapid calcium transients in astrocytes have used bulk application of membrane permeable (acetoxymethyl (AM) esters), single wavelength calcium indicators such as Rhod-2- AM or Oregon Green BAPTA-1-AM (OGB1-AM). Rhod-2 is bright and exhibits a large change in fluorescence (100-fold or more) upon calcium binding with a Kd of ∼570  nM (medium affinity).^70^ However, Rhod-2 also loads mitochondria, leading to mixed cell compartment calcium signals. OGB-1 is bright and has a high affinity for calcium (Kd∼190  nM), which is ideal to detect small concentration changes, yet has a much smaller dynamic range compared to Rhod-2 (maximum 14-fold)^70^^,^^71^ (Fig. 1). The AM versions of these dyes load astrocytes very effectively, and neither display a significant shift in the excitation/emission spectra when binding calcium.^70^ Relative calcium measurements are performed by calculating the ratio of fluorescence during a calcium signal over the average baseline fluorescence. This technique is simple and allows comparisons across experiments that exhibit differences in dye loading or photobleaching. An important consideration for calcium imaging in astrocytes using AM dyes, however, is primarily soma and larger processes labeling occurs, leaving PAP calcium transient detection suboptimal and poorly resolved.^72^^–^^74^ To combat this, nonmembrane-permeable organic calcium dyes including Fluo-4, Fura-2, or calcium green-1 potassium salts can be directly loaded into an astrocyte via whole cell patch clamp. Fluo-4 salt is commonly used because it has relatively high affinity for calcium (∼340  nM), is very bright, and has a large dynamic range (>100-fold).^73^ Advantages of this method are improved loading of the distal fine processes and that the intracellular concentration of the dye is known.^75^ It may be important to perfuse a gap junction blocker such as carbenoxolone prior to loading to prevent diffusion and dilution of the dye throughout the greater astrocyte syncytium or use a calcium indicator conjugated to dextran chains to increase the molecular weight, also preventing gap junction transport.^52^^,^^61^^,^^76^

Nevertheless, both bulk and patch loading of these calcium indicators requires coloading a secondary fluorescent probe to identify astrocytes. Typically, the dye sulforhodamine 101 (SR101) is selected for its preferential uptake by astrocytes and forgiving loading conditions. The deep-red emission of SR101 can be detected with minimal spectral overlap with GFP or green fluorescein-based calcium indicators.^77^ SR101 uptake is animal age-dependent, however,^78^ and is not equivalent in all brain regions.^79^ Some studies also show at concentrations somewhat higher than that required to label astrocytes, SR101 can cause increased neuronal excitability and long-term potentiation,^80^^,^^81^ which is an important consideration if conducting functional studies. A popular alternative approach is to express enhanced green or yellow fluorescent protein (eGFP eYFP) or red fluorescent protein such as mCherry or tdTomato in specific cellular compartments or cell types using either using AAV or a transgenic mouse line. Common astrocyte-specific promoters used are GFAP, GfaABC1D, ALDH1L1, S100β, glutamine synthetase, GLAST, and GLT-1.^82^^–^^86^ However, even when a small molecule calcium indicator is combined with a cytosolic fluorescent protein of a different color to mark morphology, views of the fine astrocyte arbor are still inadequate for determining the precise location of calcium signals (see Fig. 1). This is where the development of genetically encoded fluorescent calcium indicators (GECIs) and improved optical imaging techniques have begun to enable new observations and bring insights to astrocyte calcium signaling.

The past decade has seen significant advances in the development of GECIs. GECI expression in astrocytes is now routinely implemented from the culture dish to *in vivo*. This technology overcomes the previous requirement of loading exogenous probes and prevents any indiscriminate fluorescence labeling in tissues, cells, and even organelles that are not of interest. Indeed, GECIs expression can be localized to distinct cellular, even subcellular targets (cytosol, plasma membrane, ER membrane, mitochondria inner or outer membrane, etc.).^70^ Thus, more precise information about compartment-specific calcium dynamics can be obtained compared to highly mobile small-molecule calcium indicators that are usually limited to the cytosol but can sometimes be taken up by organelles^87^ (see Fig. 1). The ubiquitous GECI for detecting calcium signals is the single-fluorophore, intensiometric sensor GCaMP.^88^ GCaMP consists of circularly permutated green fluorescent protein (GFP) fused to calmodulin (CaM) via a fragment of myosin light chain kinase (M13). Binding of calcium causes the M13 and CaM domains to interact, limiting proton access to the internal chromophore and producing an increase in fluorescence. Many iterations of improvement have been made to the original GCaMP sensor,^88^^,^^89^ with the most widely adopted version being GCaMP6, which has three forms (slow, medium, and fast). GCaMP6 has been reported to outperform synthetic indicator dyes in terms of dynamic range.^90^ Continual development of GECIs with different calcium binding affinities ($K_{d}$), spectral properties, improved brightness, and signal-to-noise ratios, as well as GECIs that provide effective quantitative measures of calcium concentration, will further expand the ability to study calcium in astrocytes and other cells. Below, we highlight examples of GECI targeting in astrocytes.

The toolkit of GECIs to study calcium transients in astrocytes is becoming increasingly diverse and powerful. This is in part due to the ability to not only target specific cell types but also to target subcellular structures. Initial application of targeted astrocyte GECIs began with Bal Khakh’s group in 2013 who compared Lck (membrane-tethered) and cytosolic GCaMP3 *ex vivo*. The authors found both GECIs allow for effective calcium imaging throughout the entire astrocyte territory (including endfeet), which was an improvement from organic dyes. However, Lck-GCaMP3 provided a higher signal-to-noise ratio for membrane events enabling the detection of small and rapid, possibly synaptic mediated events.^91^ This was corroborated by Stobart et al. who revealed that plasma membrane calcium transients, as reported by Lck-GCaMP6, occur shortly after neuronal events (within ∼120  ms). Here, the use of targeted GECIs was crucial in the dissociating rapid plasma membrane-based transients from slower onset, IP_3_R mediated, calcium events.^39^ Earlier, Agarwal et al. used an analogous approach where they visualized GFP tethered to mitochondria in astrocytes using a mitochondrial targeting amino acid sequence and combined this with the astrocyte selective expression of GCaMP3 (*GLAST-mGC3;mito-EGF)*.^40^ This allowed them to discover that a significant fraction of astrocyte microdomain calcium signals colocalized with mitochondria. With IP_3_ signaling and mitochondria activity playing an important role in astrocyte calcium signals, use of intraorganelle GECIs is an exciting avenue for investigation.^92^ Indeed, Okubo recently used G-CEPIA1er, a GECI targeted to the ER in astrocytes, with the sensor facing inside the ER lumen. This unique tool allowed the authors to describe an IP_3_R2-independent signal in IP_3_R2–/– mice. In response to norepinephrine, which evokes large cytosolic calcium elevations in astrocytes via store calcium release, a significant fraction of the ER calcium drop remained in the absence of IP_3_R2.^93^ Recently, Haidey et al. performed spatiotemporal analysis of the endfoot calcium signals in brain slices measured via membrane anchored GCaMP6f. This Lck-GCaMP6f provided high signal-to-noise measures of calcium events localized to the endfoot-vascular membrane. They found basal endfoot calcium transients became more frequent and larger in response to vasoconstriction.^61^ In comparison, using patch loaded Fluo-4 revealed a plateau-like calcium elevation in astrocyte endfeet to the same vasoconstriction (see Fig. 1). This clearly showed how different calcium indicators report the same calcium event differently. The use of subcellular targeted GECIs in astrocytes is still in its infancy, meaning there are many opportunities for discovery. Further work investigating calcium signals from different compartments of the mitochondria in particular could reveal important insights linking metabolism and the ability of astrocytes to feed synapses or control local blood flow.

## 6 Imaging Calcium Dynamics in Astrocytes

**Fig. 2:**
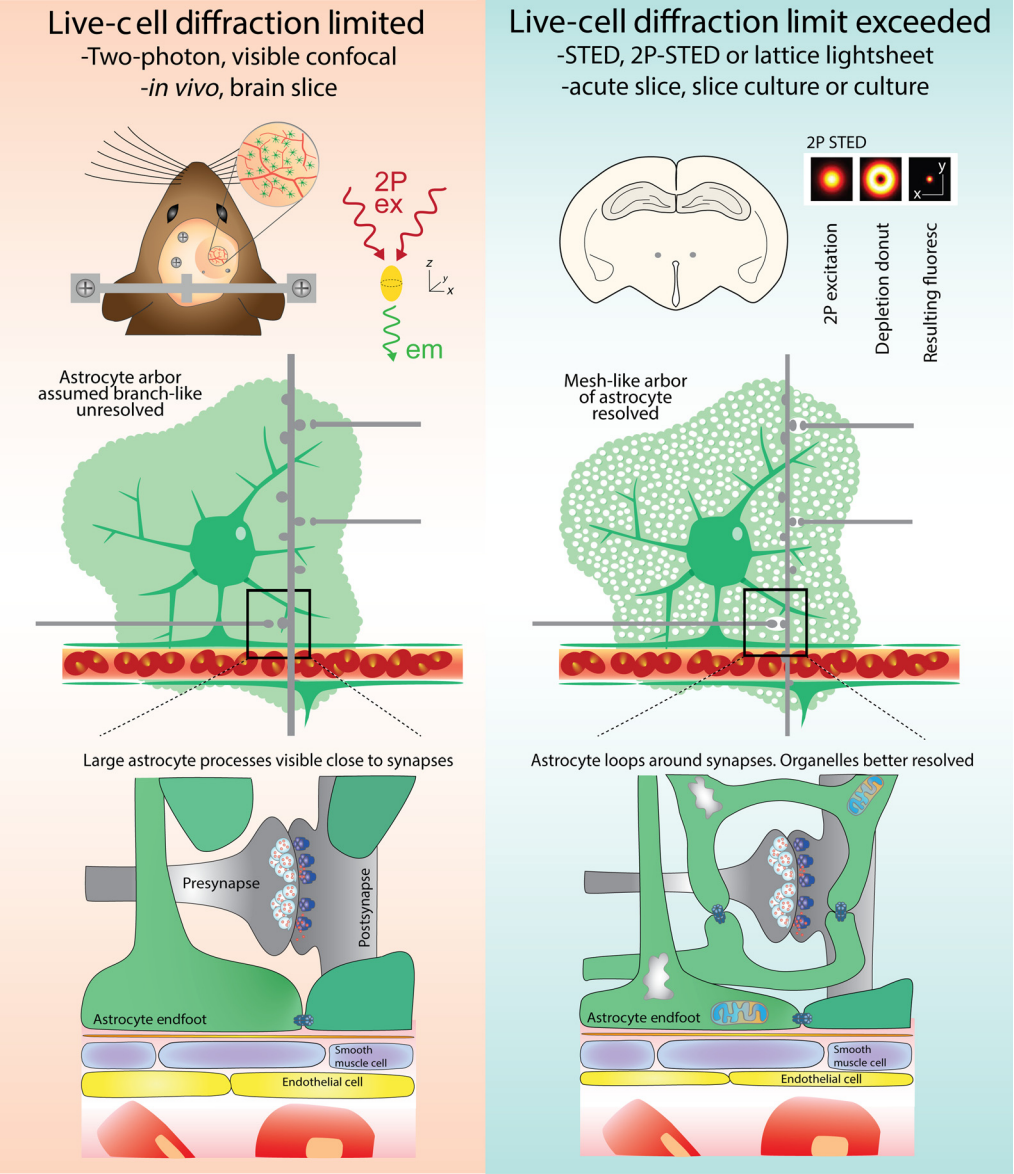
A schematic comparison of the neuro–glio–vascular interface as observed under diffraction-limited versus diffraction-limit exceeded microscopy for live-cell imaging. Diffraction limited tools (two-photon, visible confocal) are well suited to *in vivo* and brain slice preparations; however, only large astrocyte processes near synapses are resolved. Diffraction-limit exceeded microscopy (STED, 2P-STED, etc.) in acute slices or culture allow for specific compartments or organelles in addition to the loop-like/spongiform astrocyte arbor to be visualized.

Despite dramatic improvements in calcium probes, the experimental results obtained remain constrained by the imaging modalities used. For *in vivo* imaging, two-photon laser scanning microscopy (2PLSM) has become the preferred tool, as good resolution can be acquired at substantial tissue depths (>500  μm).^94^^,^^95^ 2PLSM requires simultaneous excitation of fluorophores by two long-wavelength photons, which are less susceptible to scattering throughout the tissue. This allows for increased penetration depth, reduced photobleaching, and the photons detected come from fluorophore emission exclusively at the focal plane.^96^ Laser scanning confocal microscopy (LSCM), which employs single-photon short wavelength excitation, is more sensitive to scattering and thus is better suited to imaging more superficial regions (maximum ∼50  μm from the surface).^97^ This approach utilizes a pinhole to reject fluorescence from out-of-plane sources to improve the resolution of images. Despite both methods being limited by the diffraction of light, the use of shorter wavelengths in LSCM results in superior resolution to 2PLSM as the point spread function or pattern of diffracted light from a point source is smaller (LSCM: ∼180  nm in xy axis; ∼500  nm in z axis versus 2PLSM: ∼385  nm in xy axis; ∼1500  nm in z axis).^98^^–^^101^ Given a relatively small point spread function, decreased susceptibility to light scattering and less tissue damage, 2PLSM has been a tool of choice for high resolution fluorescence imaging *in vivo*, including astrocyte work, whereas LSCM can more easily be employed for live acute slice work. However, 2PLSM is still more commonly utilized in the *ex vivo* preparation too (see Fig. 2). Using 2PLSM *in vivo* has revealed new information regarding the effects of different neurotransmitters on microdomain astrocyte calcium transients, where glutamate caused sparse and localized increases in transients, and noradrenaline caused a cell-wide elevation.^38^^,^^40^

Stimulation emission depletion (STED) microscopy is a powerful super-resolution imaging tool that strikes an appealing compromise between spatial resolution, image acquisition speed, depth, and signal-to-noise ratio, making it particularly well suited to image the fine processes of astrocytes in living tissue.^102^^,^^103^ Similar to LSCM and 2PLSM, STED is also a laser scanning method; however, it is not constrained by the diffraction limit of light.^104^ This is accomplished by the addition of a second, redshifted, doughnut-shaped laser that precisely encircles the regular excitation laser, thereby depleting the fluorescent molecules everywhere except the central region of the excitation point spread function. This can also be used in the z-axis resulting in super-resolution in all three axes (50 nm in xy axis; 250 nm in z axis)^105^ (see Fig. 2). STED microscopy can be used when labeling of the interstitial fluid using a membrane-impermeable dye in a method known as super-resolution shadow imaging (SUSHI). Upon image acquisition, all cellular structures cast a dark “shadow” and instead the extracellular space is illuminated in super-resolution. SUSHI can be combined with a positive fluorescent label of a different color in a specific cell or cell-type of interest, meaning anatomical relationships within the extracellular space can be compared in great detail.^105^

STED microscopy has provided fascinating insights into the nanostructure of the astrocyte arbor. A study by Arizono et al.^26^ found using 3D-STED in combination with confocal calcium imaging that many fine processes connect to form loops near synapses. As such, the morphology of the astrocyte arbor is more similar to a sponge that covers a given territory rather than simply linear processes extending from the soma (see Fig. 2). Further analysis of the spatial relationship between PAPs and excitatory synapses revealed that most localized calcium transients occur in compartmentalized nodes (0.07 to $0.7\;\mu m^{2}$), some of which contain organelles.^103^ Correlations between PAP and dendritic spine size, as well as between calcium transient area and spine size were reported suggesting a structural and functional link between PAPs and synapses.^26^ Similarly, it was observed using STED that postsynaptic densities and nodes of Ranvier in the spinal cord associated with astrocyte processes (forming a tripartite synapse) are more structurally complex and contain more diverse arrays of molecular machinery (signaling enzymes, neurotransmitter receptors, etc.).^106^^,^^107^

Several groups are currently examining how these microstructures are remodeled under changes in physiological state. As previously mentioned, PAPs withdraw from synapses that have recently undergone LTP induction, which promotes glutamate spillover to extra-synaptic sites.^65^ Increased neuronal activity has also been shown to drive increased expression of gap junction forming protein, connexin 30 (Cx30), on the membrane of PAPs as visualized via STED.^108^ Upregulation of Cx30 is thought to initiate morphological changes in the astrocyte thereby promoting enhanced glutamate uptake.^108^^,^^109^ Recently, SUSHI imaging revealed these loop like PAPs enclose dendrites and interstitial fluid creating a secluded microenvironment.^110^ After applying an osmotic stress to the tissue, PAPs and dendrites swell significantly, increasing the contact of these structures while decreasing the volume of interstitial fluid found in the extracellular space. Kaminski’s group has examined the link between cytoskeletal (actin/microtubule) organization on membrane properties using STED and atomic force microscopy in migrating astrocytes.^111^ However, this has not been performed in PAPs undergoing structural changes in response to physiologically relevant stimuli. Investigating cytoskeletal modifications and the influence on membrane properties in this context poses an exciting area of possible study likely contributing to neuro–glia communication.

Although there are numerous exciting applications for STED microscopy, it is important to be cognizant of limitations. While STED provides remarkable super-resolution images of live cell morphology, it is not well suited to capturing astrocyte calcium transients. This can be overcome by mapping confocal calcium images onto STED structural images of PAPs.^26^ Another consideration for imaging is that the intensity of the STED (depletion) laser is up to three orders of magnitude greater than the typical excitation laser; therefore, phototoxicity and bleaching are serious concerns.^103^ Several studies have mitigated this issue while still effectively labeling PAPs by patch loading a bright, stable dye (Alexa Fluor 488) directly^112^ or via robust transgenic expression of fluorescent protein ZsGreen.^26^ Image acquisition parameters (laser power, frame rate, and field of view) are important to optimize to further manage phototoxicity and bleaching.^103^ Lastly, the majority of recent STED studies are performed using organotypic slices, which have many advantages in terms of image acquisition/quality. However, this tissue preparation approach is limited if interested in understanding *in vivo* phenomena or the neurovascular interface. Without blood perfusion, the vascular system collapses; therefore, it is imperative to consider in which contexts STED imaging with organotypic tissue preparation is best applied.

Another interesting optical imaging method that may have utility in capturing ultrafast calcium transients is light sheet fluorescence microscopy (LSFM). A light sheet of micron-scale thickness can be generated from continuous wave lasers.^113^^,^^114^ Aligning the light sheet orthogonally to the focal plane of an objective lens enables scanless wide-field optical sectioning and ultrafast image acquisition.^115^^–^^117^ Compared to LSFM, conventional point to point scanning methods (LSCM or 2PLSM) are limited in image acquisition speed.^118^ A recent study by Pham et al. demonstrated LSFM as useful tool for mapping calcium microdomains in acute brain slices and interestingly, uncovered heterogeneity in the kinetics of spontaneous calcium transients found in cortical versus hippocampal astrocytes.^118^ Despite the speed advantages of this method, the contrast and confocality of LSFM in brain slices appears inferior to two-photon, but there may be room for improvements in sample preparation/presentation within the microscope. Furthermore, LSFM is still a diffraction-limited approach therefore it is imperative to tailor the imaging tool used to ensure it matches the biological question of interest. Nevertheless, more recent lattice lightsheet microscopy breaks the diffraction barrier^119^ and may be a preferred tool moving forward for high speed, super-resolution live-cell work in relatively transparent tissues/organisms.^119^^–^^121^

## 7 Analysis Tools for Calcium Imaging Data

Analyzing calcium transients, especially in the astrocyte arbor, is a particularly complex task. These events are numerous and dynamic in time and space, meaning they are not well suited to conventional image analysis methods. Two main strategies to analyze astrocyte calcium imaging data have emerged: (1) region of interest (ROI)-based analysis where calcium transients are treated as individual units in space and (2) event-based analysis where calcium transients are recognized as distinct events in time that are not spatially restricted. When determining which of these approaches are most effective, one should consider the biological question of interest and what information the image analysis is able to provide.

An early attempt to improve the analysis of astrocyte microdomain calcium came from Srinivasan et al., which published the open-source software GECIquant in 2015. GECIquant detects, in a semiautomated fashion, ROIs containing calcium changes above a threshold value.^38^ The software differentiates between somatic calcium fluctuations, waves moving through processes, and punctate signals in microdomains. The program provides the raw fluorescence data over time from each of the detected ROIs. Traces obtained through GECIquant can be further processed via a separate MATLAB script to obtain additional features such as the amplitude and frequency for each ROI detected.^48^

Agarwal et al. developed CaSCaDe, which uses machine-learning to identify calcium events. Thresholding values are used to isolate microdomains that can be further refined based on additional criteria of amplitude and duration. Active regions are plotted onto a spatial map and relevant descriptors (frequency, amplitude, and timing of events) can be extracted.^40^

The development of the program AQuA in 2019 by Poskanzer’s group provided additional improvements for astrocyte calcium analysis. AQuA is a departure from ROI-based analysis in favor of an event-based approach. The framework of the ROI is limiting in the context of calcium dynamics as there is an assumption that the ROI does not change shape, position, or overlap with other ROIs.^122^ In AQuA, each increase in fluorescent intensity is captured as a unique event that can be analyzed for dynamic properties such as size, shape, propagation direction, duration, frequency, and amplitude. The distinct events are identified through a variety of processing steps, including thresholding, smoothing, and the definition of spatial and temporal boundaries. Throughout the analysis process, the user can inspect traces and modify parameters to ensure optimal analysis. A valuable feature is the ability to choose between different data type presets, depending on the calcium indicator used and on the acquisition signal-to-noise ratio.^122^ Haidey et al. applied AQuA analysis of astrocyte endfoot calcium dynamics in brain slices, to reveal that vasoconstriction did not evoke a new type of calcium transient not previously present, but instead intensified existing spontaneous calcium transients as vessel diameter decreased.^61^ AQuA has also been used to analyze spontaneous astrocyte calcium signaling in *Drosophila* by the Freeman group. AQuA helped uncover a distinctive subset of spontaneous transients that are activated by local ROS production acting on TRP channel TrmPL, ultimately resulting in changes in oxygen delivery.^122^^,^^123^

Integrating complex calcium imaging with electrophysiological or behavioral datasets is a challenge that is addressed by MATLAB toolbox Begonia developed by Bjørnstad et al.. Similar to AQuA, Begonia uses an event-based approach (known as regions of activity or ROAs) to automatically detect calcium signals; however, hand-drawn ROIs can also be incorporated. Contrary to AQuA, however, Begonia allows for easy integration of other time-series data and is optimized for handling large, high-frame-rate (∼30  Hz) datasets, thereby avoiding significantly long processing times.^124^

Majority of the aforementioned analysis packages are targeted to single cell analysis. Software package Astral is designed to analyze astrocyte–astrocyte or astrocyte–neuron interactions at a network level. Astral uses an event based, thresholding method to not only measure parameters of single calcium events (duration, direction, etc.) but also evaluates intercellular propagation of calcium events between neighbors in astrocytic networks.^125^

Another useful image analysis package that has been tailored to suit astrocyte calcium imaging datasets is known as CHIPs, which is a MATLAB-based, open-source toolbox that offers the ability to combine multiple analysis approaches into a single pipeline.^126^ The flexible framework of CHIPs is ideal for examining a dataset in a variety of ways and for developers who are looking to further customize the analysis process. See Table 1 for a comparison of all previously mentioned astrocyte image analysis toolboxes.

**Table 1:** A comparison of the most current image analysis toolboxes catered to astrocyte calcium imaging data. The primary reference for each toolbox is provided along with the platform on which the toolbox is run. The strategy of analysis the toolbox preforms is also indicated (ROI versus event-based) with intended use scenarios and the key outputs generated.

Toolbox	Primary reference	Platform	ROI based	Event based	Intended uses	Key outputs
GECIquant	Srinivasan et al. (2015)	ImageJ and MATLAB	✓		User friendly, ROI-based detection using thresholding	Intensity versus time traces for each ROI
					Best suited to examine larger/localized ${\mathrm{Ca}}^{2+}$ transients within a single cell	An additional script can be used to extract ROI features (amplitude/frequency)
CaSCaDe	Agarwal et al. (2017)	MATLAB	✓		ROI-based machine learning detection of active regions	Spatial map of detected ROIs
					Best suited to examine ${\mathrm{Ca}}^{2+}$ microdomains of a single cell	Frequency and amplitude of ROIs
AQuA	Wang et al. (2019)	MATLAB or ImageJ		✓	Flexible platform, event-based machine learning detection of individual ${\mathrm{Ca}}^{2+}$ events	Frequency, amplitude, area, and duration of ${\mathrm{Ca}}^{2+}$ events
					Best suited to examine dynamic properties of distinct ${\mathrm{Ca}}^{2+}$ transients in a cell	Propagation direction, rise/decay time, and proximity to a user-identified “landmark”
Begonia	Bjørnstad et al. (2021)	MATLAB	✓	✓	Automatic, event, and ROI-based detection plus data management toolbox	Frequency, amplitude, area, and duration of ${\mathrm{Ca}}^{2+}$ events within user-defined ROIs
					Best suited to examine ${\mathrm{Ca}}^{2+}$ transients in conjunction with other time series data	${\mathrm{Ca}}^{2+}$ event data can be combined with behavioral or electrophysiological data
Astral	Dzyubenko et al. (2021)	Apache airflow		✓	Event-based detection optimized for multiple cell scenarios	Frequency, amplitude, area, and duration of ${\mathrm{Ca}}^{2+}$ events
					Best suited to examine ${\mathrm{Ca}}^{2+}$ transients propagating throughout a network	Evaluates intracellular propagation of ${\mathrm{Ca}}^{2+}$ between neighbouring cells
CHIPs	Barrett et al. (2018)	MATLAB	✓	✓	Extensible toolbox integrating preprocessing and several analysis methods	${\mathrm{Ca}}^{2+}$ event properties: frequency, amplitude, area, and duration
					Best suited to examine a dataset using multiple approaches simultaneously	E.g., can simultaneously analyze cell volume or diameter of vasculature

## 8 Concluding Remarks

The investigation of astrocytes and their complicated calcium dynamics provides a rich and intriguing area of study in the neurosciences that is undergoing constant development. Our fundamental understanding of the brain has been revolutionized as we continue to recognize the diverse functions of these cells. An overwhelming degree of complexity has emerged, particularly with regard to how astrocytes use calcium signals to orchestrate their many influences on neurons, the vasculature, and other cells. Improved experimental strategies, coupled with the technological advances described here, will provide scientists with the necessary tools to help elucidate the important role of these fascinating cells in neurophysiology.
